# PEnBayes: A Multi-Layered Ensemble Approach for Learning Bayesian Network Structure from Big Data

**DOI:** 10.3390/s19204400

**Published:** 2019-10-11

**Authors:** Yan Tang, Jianwu Wang, Mai Nguyen, Ilkay Altintas

**Affiliations:** 1 Data Science and Knowledge Engineering Laboratory, College of Computer and Information, Hohai University, Nanjing 210036, China; 2 Department of Information Systems, University of Maryland, Baltimore County, Baltimore, MD 21250, USA; 3 San Diego Supercomputer Center, University of California, San Diego, La Jolla, CA 92093, USA; mhnguyen@alumni.ucsd.edu (M.N.); altintas@sdsc.edu (I.A.)

**Keywords:** Bayesian network learning, big data, ensemble method, Distributed Data Parallelization, scientific workflow

## Abstract

Discovering the Bayesian network (BN) structure from big datasets containing rich causal relationships is becoming increasingly valuable for modeling and reasoning under uncertainties in many areas with big data gathered from sensors due to high volume and fast veracity. Most of the current BN structure learning algorithms have shortcomings facing big data. First, learning a BN structure from the entire big dataset is an expensive task which often ends in failure due to memory constraints. Second, it is quite difficult to select a learner from numerous BN structure learning algorithms to consistently achieve good learning accuracy. Lastly, there is a lack of an intelligent method that merges separately learned BN structures into a well structured BN network. To address these shortcomings, we introduce a novel parallel learning approach called PEnBayes (Parallel Ensemble-based Bayesian network learning). PEnBayes starts with an adaptive data preprocessing phase that calculates the Appropriate Learning Size and intelligently divides a big dataset for fast distributed local structure learning. Then, PEnBayes learns a collection of local BN Structures in parallel using a two-layered weighted adjacent matrix-based structure ensemble method. Lastly, PEnBayes merges the local BN Structures into a global network structure using the structure ensemble method at the global layer. For the experiment, we generate big data sets by simulating sensor data from patient monitoring, transportation, and disease diagnosis domains. The Experimental results show that PEnBayes achieves a significantly improved execution performance with more consistent and stable results compared with three baseline learning algorithms.

## 1. Introduction

A Bayesian network (BN) BBN is a probabilistic graphical model that represents a probability distribution through a directed acyclic graph (DAG) that encodes conditional dependency and independency relationships among variables in the model. BNs are widely applied to various forms of reasoning in many domains such as healthcare, bioinformatics, finance, and social services [1,2,3]. With the increasing availability of big datasets in science, government, and business, BN learning from big datasets is becoming more valuable than learning from conventional, small datasets. BN also has a widespread application in multiple-criteria decision analysis as a graphical model in Anomaly Detection [4] and Activity Recognition [5] from sensor data. However, learning BNs from big datasets requires high computational costs [6], and complexities that are not well addressed with conventional BN learning approaches. One solution is to perform the learning task in a distributed data processing and learning fashion using computation diagrams such as MapReduce [7,8]. Several research gaps still exist. It is quite challenging to select a learner from numerous BN structure learning algorithms to consistently achieve good learning accuracy. There are typically two approaches of ensemble learning for big data—algorithm level or data level. However, there is a lack of research that conducts ensemble learning at both the data level and the algorithm level. Furthermore, there is a lack of work for Bayesian Network learning integrated as part of the big data modeling and scientific workflow engine.

There are three main challenges for learning a Bayesian network from big data. First, learning a BN structure from the big dataset is an expensive task that often fails due to insufficient computation power and memory constraints. It is necessary to find an intelligent way to divide the dataset into small data slices suitable for distributed learning. Second, it is difficult to determine which BN learning algorithm would work well on a particular dataset. Lastly, the distributed learned BN structures need to be intelligently merged collectively to form a BN structure that follows the generative distribution of the big dataset.

Facing these challenges, we propose a novel approach called PEnBayes (Parallel Ensemble-based Bayesian network learning) to learn a Bayesian network from big data. The PenBayes approach consists of three phases: Data Preprocessing, Local Learning, and Global Ensemble Learning. In the Data Preprocessing phase, the entire dataset is divided into data slices for the Local Learners. We design a greedy algorithm to intelligently calculate the appropriate size of each data slice, defined as the Appropriate Learning Size (ALS). Then, the entire big dataset is divided into many data slices and sent to the Local Learners. During the Local Learning phase, a two-layered structural ensemble method is proposed to learn a Bayesian network structure from each data slice and then merge the learned networks into one local BN structure. Lastly, in the Global Ensemble Learning phase, PEnBayes uses the same structural ensemble method as in the Local Learners to merge the local BN structures into a global structure. Experimental results on datasets from three well-known Bayesian networks validate the effectiveness of PEnBayes in terms of efficiency, accuracy, and stability for learning a Bayesian network from big datasets. PEnBayes uses the entire big dataset instead of sampling data for learning.

The main contributions of this paper are as follows:

- A greedy data size calculation algorithm is proposed for adaptively partitioning a big dataset into data slices of appropriate size for distributed BN learning.
- A distributed three-layered ensemble approach called PenBayes is proposed to achieve stable and accurate Bayesian network learning from big datasets at both data and algorithm levels.
- PenBayes enables Big Data Bayesian network learning by leveraging the distributed platform [9] and the scientific workflow system [10] for advancing big data learning in the graphical model research area.

The remainder of this paper is organized as follows. In the next section, we introduce the background. Related work is in Section 3. The research problem is formalized in Section 4. Our approach is described in Section 5, with workflow integration in Section 6. Section 7 and Section 8 show experimental setups, results, and discussions. Finally, the paper is concluded in Section 9.

## 2. Background

### 2.1. Distributed Data-Parallel Patterns and Supporting Systems for Scalable Big Data Application

Several Distributed Data-Parallel (DDP) patterns, such as Map, Reduce, Match, CoGroup and Cross, have been identified to easily build efficient and scalable data parallel analysis and analytics applications [11,12,13]. DDP patterns enable programs to execute in parallel by splitting data in distributed computing environments. Originating from higher-order functional programming, each DDP pattern executes user-defined functions (UDF) in parallel over input datasets. Users only need to select the appropriate DDP pattern for their specific data processing tasks, and implement the corresponding UDFs.

Due to the increasing popularity and adoption of these DDP patterns, a number of execution engines have been implemented to support one or more of them. These DDP execution engines manage distributed resources, and execute UDF instances in parallel. When running on distributed resources, DDP engines can achieve good scalability and performance acceleration with good fault tolerance. Hadoop is the most popular MapReduce execution engine [14]. The Stratosphere/Flink system [15,16] supports five different DDP patterns. Many of the above DDP patterns are also supported by Spark operations [9]. Since each DDP execution engine defines its own API for how UDFs should be implemented, an application implemented for one engine may be difficult to run on another engine. Wang et al. gives examples on how to run binary legacy tools in parallel with partitioned datasets using the DDP framework [13].

### 2.2. Scientific Workflow System

Cloud computing evolved from the concept of utility computing, Workflows have emerged as a way to formalize and structure data analysis, thus becoming an increasingly popular paradigm for scientists to handle complex scientific processes [17]. The Kepler scientific workflow system [10] is an open-source, cross-project collaboration to serve scientists from different disciplines [18,19]. Kepler adopts an actor-oriented modeling paradigm for the design and execution of scientific workflows. Kepler has been used in a wide variety of projects to manage, process, and analyze scientific data.

Kepler provides a graphical user interface (GUI) for designing, managing and executing scientific workflows, which are a structured set of steps or tasks linked together that implement a computational solution to a scientific problem. In Kepler, Actors provide implementations of specific tasks and can be linked together via input and output Ports. Data are encapsulated in messages or Tokens, and transferred between actors through ports. Actor execution is governed by Model of Computations (MoCs), called Directors in Kepler [20].

Since each DDP pattern expresses an independent higher-order function, we can define a separate DDP actor for each pattern. Unlike normal actors, these higher-order DDP actors do not process the input data sent to them as a whole. Instead, they first divide the input data and then process each partition independently.

The UDF for the DDP patterns is an independent component and can naturally be encapsulated within a DDP actor. The logic of the UDF can either be expressed as a sub-workflow. In the first case, users can compose a sub-workflow for their UDF in the Kepler GUI using specific subsidiary actors for the DDP pattern and any other general actors. Since the sub-workflow is not specific to any engine API, the same sub-workflow could be executed on different DDP engines. Like other actors, multiple DDP actors can be linked to construct larger, more complex applications.

**Figure 1:**
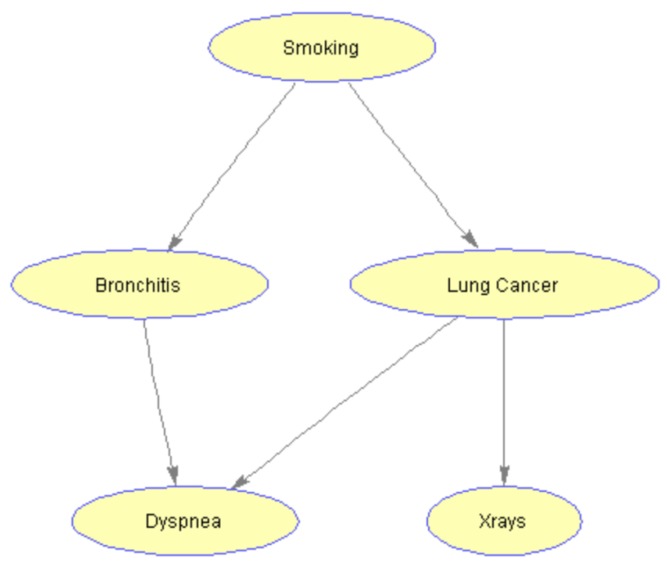
A Bayesian network example - Cancer Network.

A Bayesian network (BN) is a probabilistic graphical model that represents a probability distribution through a directed acyclic graph (DAG) that encodes conditional dependency and independency relationships among variables in the model [21]. Consider the following example in Figure 1, representing a simplified model to help diagnose the patients arriving at a respiratory clinic. This BN is known as Cancer network [22] containing five nodes: Smoking, Bronchitis, Lung cancer, Dyspnea, and X-ray. A history of smoking has a direct influence on both whether or not a patient has bronchitis and whether or not a patient has lung cancer. Therefore, in the network model, the node ‘Smoking’ is the parent of nodes ‘Bronchitis’ and ‘Lung cancer’. In turn, the presence or absence of lung cancer has a direct influence on the results of a chest X-ray test, so the node ‘Lung cancer’ is the parent of the child node ‘X-ray’ with a directed arc connecting them.

### 2.3. Bayesian Network

In Bayesian network research, the notion of *DAG-faithful* was introduced in the work of Cheng et al. [23]. A dataset is DAG-faithful if its underlying probabilistic model can be structured as a DAG. This condition makes a dataset suitable for BN learning.

**Definition** **1.** *(DAG faithfulness)*

*A graph G is a dependency map (D-map) of a probabilistic distribution P if every dependence relationship derived from G is true in P; G is an independency map (I-map) of P if every independence relationship derived from G is true in P. If G is both a D-map and an I-map of P, then G is a perfect map (P-map) of P, and P is a DAG-Isomorph of G [24]. Distribution P and DAG graph G are faithful to each other and P is a DAG-faithful distribution [25]. A dataset D is DAG-faithful if and only if its underlying probability distribution P is a DAG-faithful distribution.*

Technically, the objective is to find a graph that is a P-map of the true distribution of the big dataset. Spirtes et al. and Meek prove that both Gaussian and discrete distributions are faithful to a Bayesian network [25,26].

The *essential graph* [23] of a BN is a graph that has the same edges of the BN and the same *v-structures*. A triple of nodes *X*, *Y*, *Z* forms a *v-structures* if X→Z←Y and X is not adjacent to Y.

In a Bayesian network, the *Markov Blanket* (MB) of a node includes its parents, its children, and the children’s parents [24]. The MB of a node contains all the variables that shield the node from the rest of the network and is the only knowledge needed to predict the behavior of the node. Many algorithms like MMHC [27] were proposed to learn BN structure by discovering the MB of each node.

An important property for measuring the complexity of a BN is the Average Markov Blanket Size [28], denoted as AMBS.

**Definition** **2.** *(Average Markov Blanket Size)*

*Given a Bayesian network structure B with P nodes, its Average Markov Blanket Size (AMBS) is the sum of each node’s Markov Blanket Size $MBS_{i}$ divided by P.*

Formally, AMBS is calculated by Equation (1):

(1) $$AMBS=\sum_{i=1..N}MBS_{i}/P$$

Given a dataset *D* and a BN structure *B*, let $X=\{X_{1},\ldots ,X_{n}\}$ be the set of discrete random variables/nodes in *B*, $\pi_{i}$ be the set of parents of $X_{i}$ and $\pi =\{\pi_{1},\ldots ,\pi_{n}\}$, then the joint probability of the *B* and *D* denoted as P(B,D) is defined as

(2) $$P\left(B,D\right)=P\left(B\right)\prod_{i=1}^{n}\prod_{j=1}^{q_{i}}\frac{\Gamma \left(N_{ij}^{{}^{\prime }}\right)}{\Gamma \left(N_{ij}^{{}^{\prime }}+N_{ij}\right)}\prod_{k=1}^{r_{i}}\frac{\Gamma \left(N_{ijk}^{{}^{\prime }}+N_{ijk}\right)}{\Gamma \left(N_{ijk}^{{}^{\prime }}\right)}$$

Given a dataset *D*, $q_{i}$ is the number of unique configurations (*i.e.*, states) of $\pi_{i}$, $r_{i}$ is the number of values for $X_{i}$, $N_{ijk}$ is the number of times when $X_{i}=k$ and $\pi_{i}=j$, $N_{ij}=\sum_{k}N_{ijk}$, $N_{ijk}^{{}^{\prime }}$ is the hyperparameter for when $X_{i}=k$ and $\pi_{i}=j$, $N_{ij}^{{}^{\prime }}=\sum_{k}N_{ijk}^{{}^{\prime }}$ and Γ(x)=(x−1)! is the gamma function.

This joint probability is called the Bayesian Dirichlet equivalence with uniform prior (BDeu) score function [29]. The BDeu score function is one of several Bayesian score functions. It verifies the score equivalence property and is generally applicable when the search is carried out in the space of equivalence classes. This paper uses the BDeu score function as the Bayesian score function.

### 2.4. Bayesian Network Learning Algorithms

In this paper, we conduct ensemble learning at both data and algorithm levels.

Over the last decades, numerous algorithms have been proposed for learning a Bayesian network from data, such as Hill Climbing (HC) [29], Tabu Search (Tabu) [29], Three-Phase Dependency Analysis (TPDA) [23], Inter-IAMB [30], Max-Min Hill-Climbing (MMHC) [27], Breeding Swarm based algorithm [31], an improved heuristic equivalent search algorithm [32] and a data stream learning approach [33]. However, these BN learning algorithms operate individually, they do not consider the structures learned by other algorithms and incorporate them to obtain a more accurate and stable result.

There exists a type of method that finds the optimal solution in a search space [34]. Exact Bayesian network learning methods aim to find the optimal network structure given a dataset [35,36,37]. However, exact methods are usually associated with long execution times on small datasets [36,37]. This paper chooses to use HC, Tabu, and MMHC as basic learning algorithms.

## 3. Related Work

In the field of BN learning from big data, Chickering et al. [6] showed that identifying high-scoring BN from a large dataset is NP-hard. Yoo et al. [1] reviewed bioinformatics and statistical methods and concluded that Bayesian networks are suitable in analyzing big datasets from clinical, genomic, and environmental domains. In recent years, data parallelization techniques have become a key solution for big dataset Bayesian network learning problems. Fang et al. [7] proposed a Map-Reduce-based method for learning BN from massive datasets. Our previous work [8] adopted distributed data parallelism techniques and scientific workflow for BN learning from big datasets to achieve better scalability and accuracy. Yue et al. studied a parallel and incremental approach for D ata-Intensive BN learning [38]. A scalable approach for learning Bayesian network classifiers is proposed in [39]. It is quite difficult to select a learner from numerous BN structure learning algorithms to consistently achieve good learning accuracy. This is a research gap that needs to be filled. Furthermore, we significantly extend our previous work [8,40] in adopting different BN structure learning algorithms in the Local Learner and design a three-layered ensemble approach to ensure learning stability and accuracy. There are three major differences: (1) formalized problem definition and a new theorem are provided; (2) an additional layer of ensemble is added at the data slice level to leverage multiple BN structure learning algorithms for achieving learning stability; (3) extensive experiments and results analyses on much larger datasets on a distributed platform integrating scientific workflow are presented.

In machine learning, ensemble methods [41] use multiple learning methods to obtain better predictive performance than learning from any of the constituent methods. Because the multiple learning methods of ensemble learning can run independently before their results are merged, it is a natural fit to use the MapReduce model for ensemble learning. There are typically two approaches of ensemble learning for big data: (1) data level, where the results of the same learner on different data sets are merged in the end [42]; (2) algorithm level, where the results of different learning algorithms on the same data are merged in the end. As a data-level approach, Ref. [43] partitions the data, uses a decision tree method called C4.5 at Map phase for each data partition, and bagging ensemble learning [44] where individual learner results are assembled during the Reduce phase. As one of the first big data ensemble learning studies of a Bayesian network, our previous work [8] also uses the data-level approach because it uses the MMHC algorithm. As an algorithm-level approach, Refs. [45,46] support parallel ensemble learning of multiple classifiers on the same data. Different from these approaches, in this paper, we conduct ensemble learning at both the data level and algorithm level.

There have been machine learning libraries built on big data engines to support distributed learning. The most popular ones include Spark MLlib [47], Mahout [48], H2O [49], and FlinkML [50]. Users can build machine learning applications using these libraries and the inherent parallel execution from the underlying big data engines. However, to the best of our knowledge, there is no library built on big data modeling engines for Bayesian Network learning, this research fills this gap. Further, in contrast to these libraries, our approach allows researchers with limited knowledge programming to implement their own learning algorithms, and the parallel computation work is integrated as part of the big data modeling and workflow engine.

## 4. Problem Formulation

Our goal is to use the big training data to learn an accurate model of the underlying distribution at both data level and algorithm level to achieve better learning accuracy, stability, and usability towards integrating Bayesian network learning as part of the big data modeling and scientific workflow engine. We need to state and elaborate this more precisely using the following definitions and theorem.

Given a very large DAG-faithful dataset, it is desirable to divide it into many slices for distributed learning. The key challenge is to determine the data slice size. A size that is too small breaks the DAG-faithful property of the data slice, resulting in a poor BN structure, whereas a size that is too large may incur a high computation cost. Thus, we introduce a concept called Appropriate Learning Size (ALS), as defined below.

**Definition** **3.** *(Appropriate Learning Size)*

*Given a DAG-faithful and independent identically distributed (iid) big dataset D, its Appropriate Learning Size ($ALS_{D}$) is the minimal data slice size that maintains the DAG-faithful property.*

**Definition** **4.** *(Edge Strength) Given a dataset D containing N records and a Bayesian network B containing M edges, the Edge Strength (ES) of B is the Bayesian score of B given D divided by M and N.*

Formally, Edge Strength of *B* given *D* is calculated by Equation (3):

(3) $$ES\left(B,D\right)=\frac{P(B,D)}{\left(N*M\right)}$$

Edge Strength (ES) indicates the average contribution of each edge to the overall score of the BN. A lower ES value means higher network quality and structural stability. This paper uses the BDeu score function for Edge Strength calculation throughout the experimental study.

**Theorem** **1.** *Given a distribution P, and a sufficiently large DAG-faithful dataset $D_{S2}$ with S2 records and a DAG structure $BN_{D_{S2}}$, there exists a DAG-faithful dataset $D_{S1}$ drawn from P, with S1 records and a DAG structure $BN_{D_{S1}}$ (S1<S2). The difference of the average Markov blanket size between the $BN_{D_{S1}}$ and $BN_{D_{S2}}$ is less than a small threshold β. The absolute value difference of the Edge Strength between the $BN_{D_{S1}}$ and $BN_{D_{S2}}$ is less than a small threshold ϵ.*

*This theorem can be formalized as:*

(4) $$\begin{array}{c} \forall P,D_{S2},\exists \epsilon ,\beta ,\exists D_{S1},S1<S2|\\ AMBS\left(BN_{D_{S1}}\right)-AMBS\left(BN_{D_{S2}}\right)<\beta \\ |ES\left(BN_{D_{S1}},D_{S1}\right)-ES\left(BN_{D_{S2}},D_{S2}\right)|<\epsilon \end{array}$$

**Proof.**  By Definition 1, since $D_{S1}$ and $D_{S2}$ are both DAG-faithful datasets, their generative probability distributions *P* have unique essential graphs $G_{D_{S1}}$ and $G_{D_{S2}}$, which encodes the same conditional independences in *P* [23]. Since the essential graphs $D_{S1}$ and $D_{S2}$ are drawn from the same distribution *P*, then $G_{D_{S1}}$ and $G_{D_{S2}}$ are identical. The only difference between an essential graph and a DAG structure is the edge direction, but because the change of arc direction does not effect the sum of each node’s Markov Blanket, the change of edge direction will not affect the AMBS. Thus, we can transform $G_{D_{S1}}$ and $G_{D_{S2}}$ into two DAGs: $BN_{D_{S1}}$ and $BN_{D_{S2}}$, and consequently, $AMBS(BN_{D_{S1}})$ is equal to $AMBS(BN_{D_{S2}})$. Therefore, there must exist a threshold value β such that $AMBS\left(BN_{D_{S1}}\right)-AMBS\left(BN_{D_{S2}}\right)<\beta$.

Furthermore, since $BN_{D_{S1}}$ and $BN_{D_{S2}}$ have the same edges, the differences between $ES(BN_{D_{S1}},D_{S1})$ and $ES(BN_{D_{S2}},D_{S2})$ are determined by the Bayesian score calculation over the different orientation of edges, which is insignificant and converges towards 0 when S1 and S2 become sufficiently large [29]. Therefore, given a distribution *P*, and a sufficiently large DAG-faithful dataset $D_{S2}$ with S2 records, there exists a small threshold, ϵ, and $D_{S1}$ such that S1<S2 and $|ES\left(BN_{D_{S1}},D_{S1}\right)-ES\left(BN_{D_{S2}},D_{S2}\right)|<\epsilon$. □

Based on Theorem 1, we can propose a new greedy method that keeps increasing the size of data slice *D* until $AMBS(BN_{D})$ and $ES(BN_{D},D)$ both converge. In this way, we can quantitatively estimate ALS and verify dataset’s DAG-faithful property for distributed Bayesian network learning on a big dataset. Details of this greedy method can be found in Algorithm 1.

In practice, the underlying network structure of a dataset *D* is unknown. The only way to estimate the AMBS is through learning and obtaining the BN structure from the *D*. To address this challenge, based on Theorem 1, we propose a greedy algorithm (Algorithm 1) to estimate the value of ALS using AMBS and Edge Strength.

Algorithm 1 starts with a small data slice, $D_{sliced}$. It learns the BN from $D_{sliced}$ (Step 4) and obtains AMBS (Step 5) and Edge Strength(ES) (Step 5). Since $D_{sliced}$ may not be large enough to be DAG-faithful, resulting in inaccurate and unstable AMBS and ES. In order to make $D_{sliced}$ DAG-faithful, the loop in the algorithm (Steps 7–16) doubles sliceSize at each iteration, the loop condition ensures that currentAMBS and currentES values converge after each iteration. We choose to double the size because this is a reasonable learning rate that serves as a balanced tradeoff point between quality and efficiency. If the size increases at a lower learning rate, then it will take longer for the algorithm to converge, if the rate is a more significant number such as 3, then the ALS will grow too fast, resulting in a relatively large data slice that may slow down the learning process. The loop stops when both AMBS and ES become stable, which satisfies Theorem 1. This indicates, based on Theorem 1, that the size of $D_{sliced}$ is close to ALS (Step 17) and can approximate the real ALS value.

**Algorithm 1** CalculateALS.
**Input:**

*D*: Dataset;
*ϵ*_1_, *ϵ*_2_: Thresholds;
*mstep*: Maximum loop steps.
**Output:**

*AMBS*: Average Markov blanket size;
*ALS*: Appropriate Learning Size.1:*bestAMBS* = 1; *bestES* = −1; step = 0;2:sliceSize = InitialSize * number of attributes in *D*;// Initial data slice size3:*D_sliced_* = read sliceSize rows from D;4:*BN_DS_* = LearnBNStructure(*D_sliced_*);5:*currentAMBS* = average Markov Blanket size of *BN_DS_*;6:*currentES* = Edge Strength of *BN_DS_*;7:**while** (*step* ≤ *mstep*) AND ((|*currentAMBS* − *bestAMBS*| > *bestAMBS* * *ϵ*_1_) OR (|*currentES* −
*bestES*| > *bestES* * *ϵ*_2_)) **do**8: *sliceSize* = *sliceSize* * 2;9: *bestAMBS* = *currentAMBS*10: *bestES* = *currentES*;11: *D_sliced_* = readData(*D*, *nrows* = *sliceSize*);12: *BD_DS_* = learnBNStructure(*D_sliced_*);13: *currentAMBS* = AMBS of *BD_DS_*;14: *currentES* = Edge Strength of *BD_DS_*;15: *step* = *step* + 1;16:
**end while**
17:*ALS* = number of records in *D_sliced_*;18:**return***ALS*.

**Definition** **5.** *(Weight of Bayesian network )*

*Given M Bayesian network structures, each structure denoted as $B_{i}$, a dataset D and a Bayesian score function P on $B_{i}$ and D denoted as P(B,D), then, the weight of a structure $B_{k}$, denoted as $W(B_{k})$, is its score divided by the sum of scores of all the structures.*

Formally, $W(B_{k})$ is calculated using Equation (5):

(5) $$W(B_{k})=\frac{P(B_{k},D)}{\sum_{i=1..M}P(B_{i},D)}$$

**Definition** **6.** *(Weighted Adjacent Matrix)*

*Given N Bayesian network structures, each structure denoted as $B_{i}$, a dataset D and a score function F(B,D). The Weighted Adjacent Matrix of the structure $B_{i}$, denoted as ${\mathrm{WAM}}_{B_{i}}$, is the product of $B_{i}$’s adjacent matrix ${\mathrm{AM}}_{B_{i}}$ and $W(B_{i})$.*

Formally, ${\mathrm{WAM}}_{B_{i}}$ is calculated using Equation (6):

(6) $${\mathrm{WAM}}_{B_{i}}={\mathrm{AM}}_{B_{i}}\times W(B_{i})$$

**Definition** **7.** *(Final Weighted Adjacent Matrix)*

*Given a collection of N weight adjacent matrix ${\mathrm{WAM}}_{B_{i}},i\in \left[1..N\right]$, the final weighted adjacent matrix FWAM is their sum, denoted as:*

(7) $$\mathrm{FWAM}=\sum_{i=1..N}{\mathrm{WAM}}_{B_{i}}$$

## 5. The Proposed Approach

### 5.1. Overview of PEnBayes

**Figure 2:**
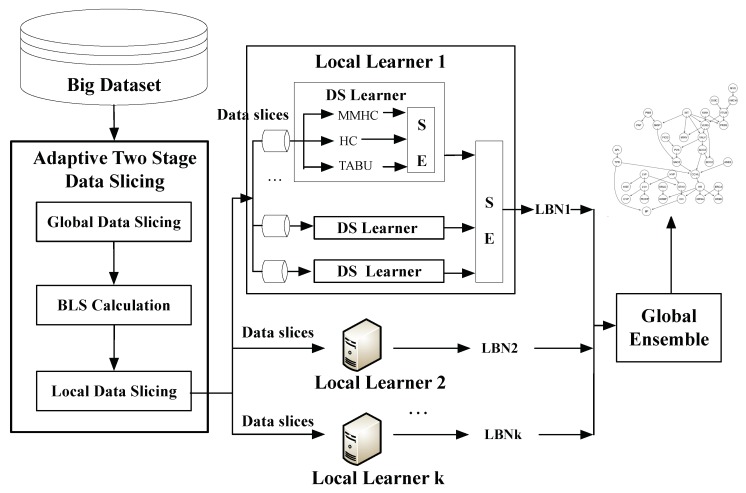
An Overview of PEnBayes approach.

Figure 2 provides an overview of PEnBayes for learning Bayesian network from big data. PEnBayes contains three phases: Data Preprocessing, Local Learner, and Global Ensemble.

#### 5.1.1. Adaptive Two-Stage Data Slicing

Given a big dataset containing *N* records and *K* local learners, the first step in Adaptive Two-Stage Data Slicing is to perform the global data slicing on the big dataset to allocate data evenly among available local learners for load balancing and consequent learning. Each local learner will receive a global data slice of N/K rows as the input for local learning. Then, the Appropriate Learning Size (ALS) value is calculated to obtain the appropriate size of each local data slice using the algorithm proposed in Section 4. The global data slice on each local learner is adaptively divided into many local data slices of size ALS for efficient and distributed learning.

Given *K* Local Learners, each local learner will receive $N_{d}$ data slices for distributed learning, each data slice is of size ALS. $N_{d}$ calculated by Equation (8).

(8) $$N_{d}=\frac{N}{K*ALS}$$

#### 5.1.2. Local Learner

Each Local Learner has two components: Data Slice Learner and Local ensemble.

Each Data Slice Learner (DS learner) learns *K* BN structures from one data slice using *K* different Bayesian network structure learning algorithms. In the experiment, three BN structure learning algorithms are used, namely MMHC, HC, and Tabu. Therefore, the value of *K* equals 3. Each DS learner then uses the first layer of the weighted adjacent matrix based structure ensemble method to combine these BN structures into one BN.

Then, the Local Learner uses the second layer of the Structure Ensemble to merge the networks learned from all data slices into one local network. As a result, each Local Learner produces one local BN. Our ensemble method is a bagging strategy based on weighted voting.

#### 5.1.3. Global Ensemble

In this final stage, upon the completion of all Local Learners, PEnBayes uses the third layer of the ensemble method to merge the network structures learned from each Local Learner into one global BN structure, thus achieving the task of learning a complete BN from a big dataset.

### 5.2. Structure Ensemble Method

The main reasoning for the Structure Ensemble Method (Algorithm 2) is based on Definition 5 and Definition 7. Given *N* BN structures and a dataset *D*, the goal is to merge these structures into one network structure $BN_{E}$. Edge Strength is used as the score function $F(B_{k},D)$ to indicate the quality of a learned structure among all the structures (Definition 5). Then, by modeling the learned structures as the Final Weighted Adjacent Matrix FWAM (Definition 7), an edge *e* should exist in the $BN_{E}$ when *e* exists in the majority of the learned structures. This is equivalent to the the formalized condition FWAM[i,j]>γ in which γ is the Structure Ensemble Threshold.

**Algorithm 2** StructureEnsemble.
**Input:**

*BN*: BN Structures;
D: Data set;
*T*: Threshold factor.
**Output:**

*BN_E_*: Ensembled BN Structure.1:Obtain AM[i] from $BN_{i}$;2:ES[i] = $ES(BN_{i},D)$;3:$W(BN_{i})$ = ES[i]/∑ES[k]4:${\mathrm{WAM}}_{BN_{i}}$ = $\mathrm{AM}[i]*\;W(BN_{i})$;5:$\mathrm{FWAM}=\sum {\mathrm{WAM}}_{BN_{i}}$;6:$\gamma =T*min(W\left(BN_{i}\right))$,7:**if**FWAM[i,j]>γ and i->j does not form a circle in $BN_{E}$
**then**8: $BN_{E}\left[i,j\right]$ = 1;9:
**end if**
10:**return**$BN_{E}$.

Specifically, the Structure Ensemble Method works as follows: First, the Structure Ensemble encodes each network $BN_{i}$ into an adjacent matrix AM[i], then, it calculates the weight of each network using Edge Strength as the score function, and obtains the weighted adjacent matrix using Equation (6) (Steps 2–4). In Step 5, the final weighted adjacent matrix FWAM is obtained. FWAM[i,j] is the collective voting results of the structures with respect to their weights and is calculated using Equation (7). Based on majority voting, if a directed edge exists between node *i* and node *j* in most of the networks, then FWAM[i,j] should be larger than the Structure Ensemble Threshold γ Equation (9). The basic threshold is equal to the minimal structure weight $min(W\left(B_{i}\right))$. Given that *T* is the basic threshold factor, if an entry FWAM[i,j] is larger than the threshold $T*min(W\left(B_{i}\right))$, then there are more than *T* structures containing the edge j→i. Therefore, the Structure Ensemble adds an edge between *i* and *j* in the network $BN_{E}$ (Step 8). After iterating all the entries in FWAM, a merged network structure $BN_{E}$ is produced from BN.

(9) $$\gamma =T*min(W\left(B_{i}\right))$$

For example, suppose there is a dataset *D* from the Cancer network, and we learned three different networks $B_{1}$, $B_{2}$ and $B_{3}$ from *D* with the following Adjacent Matrix.

$$B_{1}=\left[\begin{array}{ccccc} 0 & 0 & 0 & 0 & 0\\ 1 & 0 & 0 & 0 & 0\\ 1 & 0 & 0 & 0 & 0\\ 0 & 1 & 0 & 0 & 0\\ 0 & 0 & 1 & 0 & 0 \end{array}\right]\;B_{2}=\left[\begin{array}{ccccc} 0 & 0 & 0 & 0 & 0\\ 1 & 0 & 0 & 0 & 0\\ 1 & 0 & 0 & 0 & 0\\ 0 & 0 & 1 & 0 & 0\\ 0 & 0 & 1 & 0 & 0 \end{array}\right]\;B_{3}=\left[\begin{array}{ccccc} 0 & 0 & 0 & 0 & 0\\ 1 & 0 & 0 & 0 & 0\\ 1 & 0 & 0 & 0 & 0\\ 0 & 1 & 1 & 0 & 0\\ 0 & 0 & 0 & 0 & 0 \end{array}\right]$$

The Structure ensemble calculates the Edge Strength of each network $B_{i}$ given *D* and uses Equation (5) to calculate the $W(B_{i})$. In this case, we have $W(B_{1})$ = 0.31, $W(B_{2})$ = 0.34, $W(B_{3})$ = 0.35. Then,

$$\begin{array}{c} \;\mathrm{FWAM}=\left[\begin{array}{ccccc} 0 & 0 & 0 & 0 & 0\\ 0.31 & 0 & 0 & 0 & 0\\ 0.31 & 0 & 0 & 0 & 0\\ 0 & 0.31 & 0 & 0 & 0\\ 0 & 0 & 0.31 & 0 & 0 \end{array}\right]+\left[\begin{array}{ccccc} 0 & 0 & 0 & 0 & 0\\ 0.34 & 0 & 0 & 0 & 0\\ 0.34 & 0 & 0 & 0 & 0\\ 0 & 0 & 0.34 & 0 & 0\\ 0 & 0 & 0.34 & 0 & 0 \end{array}\right]+\\ \left[\begin{array}{ccccc} 0 & 0 & 0 & 0 & 0\\ 0.35 & 0 & 0 & 0 & 0\\ 0.35 & 0 & 0 & 0 & 0\\ 0 & 0.35 & 0.35 & 0 & 0\\ 0 & 0 & 0 & 0 & 0 \end{array}\right]=\left[\begin{array}{ccccc} 0 & 0 & 0 & 0 & 0\\ 1.0 & 0 & 0 & 0 & 0\\ 1.0 & 0 & 0 & 0 & 0\\ 0 & 0.66 & 0.69 & 0 & 0\\ 0 & 0 & 0.65 & 0 & 0 \end{array}\right] \end{array}$$

If we set the basic threshold factor *T* as 2, then the Structure Ensemble Threshold $\gamma =2*min(W\left(BN_{i}\right))$ = 0.62, the merged network structure is as follows: $BN_{E}$ = $\left[\begin{array}{ccccc} 0 & 0 & 0 & 0 & 0\\ 1 & 0 & 0 & 0 & 0\\ 1 & 0 & 0 & 0 & 0\\ 0 & 1 & 1 & 0 & 0\\ 0 & 0 & 1 & 0 & 0 \end{array}\right]$.

$BN_{E}$ is the correct adjacent matrix presentation of the Cancer network. Through this example, we can see that even though $B_{1}$, $B_{2}$ and $B_{3}$ all miss one edge, the Structure Ensemble can identify all five directed edges and obtain the correct network structure of the Cancer network.

### 5.3. Data Slice Learner

Data Slice Learner is the first layer of the ensemble method that executes Algorithm 3 to combine BNs learned by different learning algorithms using majority voting. Algorithm 3 invokes three BN learning algorithms (namely MMHC, HC, and Tabu) to learn a BN structure for each data slice DS. Then, it calls Structureensemble to merge the three learned networks into one network structure $BN_{DS}$. For Data Slice Learner, the basic threshold factor *T* is set as two for majority voting.

**Algorithm 3** DataSliceLearner.
**Input:**

*DS*: Data slice.
**Output:**

*BN_DS_*: Merged network structure in matrix.1:$BN_{MMHC}$ = MMHC(DS);2:$BN_{HC}$ = HC(DS);3:$BN_{Tabu}$ = Tabu(DS);4:
$BNs=[BN_{MMHC},BN_{HC},BN_{Tabu}]$
5:
T=2;
6:$BN_{DS}=StructureEnsemble\left(BNs,DS,T\right)$;7:
**return**
$BN_{DS}$


### 5.4. Local Learner

Each Local Learner (shown in Algorithm 4) is given $N_{d}$ data slices Equation (8). Then, the Local Learner learns a network for each data slice using DataSliceLearner (Step 3). The Local Learner keeps track of the data slice $DS_{B}$ with the best Edge Strength. After completing data slice learning, the Local Learner calls the Structureensemble to merge the learned networks into one local network $BN_{local}$. By Definition 5, the weight of each learned network is calculated using one dataset. For the Local Learner, the threshold factor $T=N_{d}/2$, this means the Local Learner identifies an edge if that edge exists in more than half of networks learned by the Data Slice Learners. Local Learner is the second layer of the network ensemble scheme.

**Algorithm 4** LocalLearner.
**Input:**

*DS*: Data slices;
*N_d_*: number of data slices.
**Output:**

*BN_Local_*: Local network structure.1:
**For each**
$DS_{k}$
2:$BN_{DS}\left[k\right]=\mathrm{DataSliceLearner}(DS_{k})$;3:$DS_{B}$ = the data slice with the best Edge Strength;4:
**End For**
5:
$BN_{local}=Structureensemble\left(BN_{DS},DS_{B},N_{d}/2\right)$
6:**return**$BN_{local}$.

### 5.5. Global Ensemble

Upon the completion of all the Local Learners, each Local Learner sends its local BN and its best data slice to the Global Ensemble for the final merging process (shown in Algorithm 5). Similar to the Local Learner, the third layer of the network ensemble is executed to produce one final network structure $BN_{final}$ using all the local structures and a data slice (denoted as $DS_{BG}$) as representative for the entire dataset for fast calculation here. For the Global Ensemble, given *K* Local Learners, the threshold factor is T=K∗2/3. This means that the Global Ensemble adds an edge in the final network $BN_{final}$ if that edge exists in more than two-thirds of networks learned by all the Local Learners.

**Algorithm 5** GlobalEnsemble.
**Input:**

*LS*: Local Structures;
*DS_BG_*: Data slice with the best global Edge Strength;
*K*: Number of Local Learners.
**Output:**

*BN_final_*: Local network structure.1:
$BN_{final}=StructureEnsemble\left(LS,DS_{BG},2*K/3\right)$
2:**return**$BN_{final}$.

### 5.6. The Time Complexity of PenBayes

Since PEnBayes is a parallel learning algorithm, the time complexity is determined by the local learner. As specified in Section 5.1, each local learner receives $N_{d}$ data slices. Therefore, the time complexity of PEnBayes is defined as:

(10) $$N_{d}*T\left(DS\right),$$

where T(DS) is the time spent on learning the BN structure from one data slice of size ALS and can be regarded as a constant value given the learning algorithms in the Data Slice learner and the data slice. Therefore, the time complexity of PEnBayes is linear with the value of $N_{d}$. The learning time will decrease with more local learners and will increase with fewer local learners. The experiment results in Section 8.3 confirm this theoretical analysis.

## 6. PEnBayes Workflow in Kepler

### 6.1. Overall Workflow

**Figure 3:**
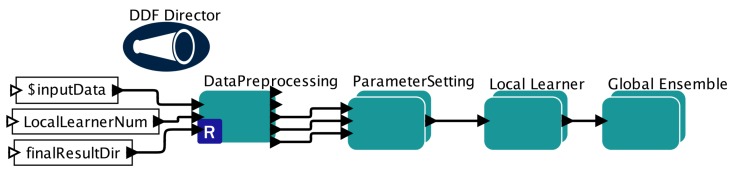
Full PEnBayes Workflow in Kepler.

We build our PEnBayes workflow by embedding the components in Section 5 into the Kepler workflow system [10]. The overall workflow is shown in Figure 3. Following the phases explained in Section 5, the workflow has three main sub-workflows: namely Data Preprocessing, Local Learner, and Global Ensemble. We use the DDF Director for Dynamic Dataflow execution semantics because the workflow has branching control structures.

### 6.2. ALS Calculation Sub-Workflow

**Figure 4:**
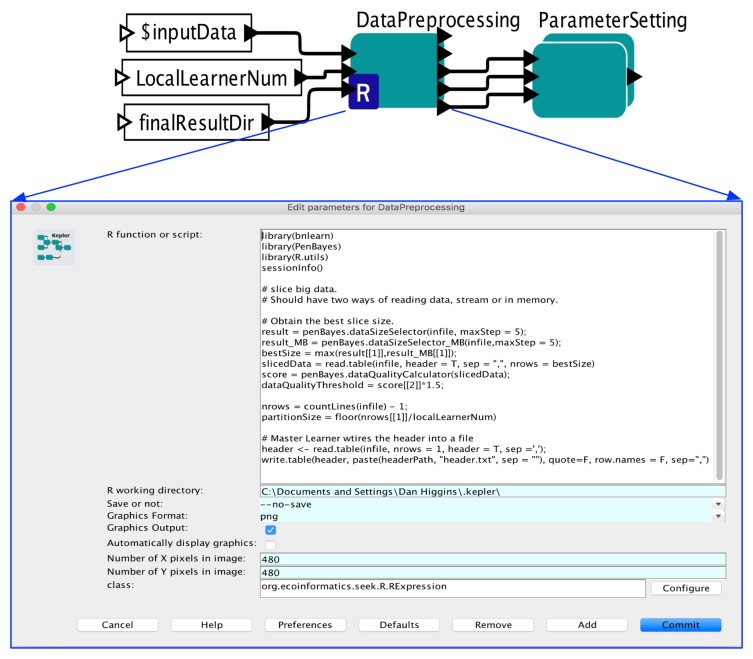
Data Preprocessing Sub-workflow.

Figure 4 shows how the Data Preprocessing is implemented in Kepler. The core component here is the DataPreprocessing actor which is an RExpression Actor to run R programs, where the R program is shown in the lower part of the figure. Algorithm 1 is implemented in R and embedded in this actor. The dark triangles on the left of the actors are used to feed parameters of the algorithm so that users can specify parameter values via the Kepler GUI or command line settings. The outputs of Algorithm 1 are fed to its downstream actor, called ParameterSetting. This actor sets values to global parameters like the Appropriate Learning Size (Definition 3), so that the calculated values can be used in Local Learner and Global Ensemble sub-workflows.

### 6.3. Local Learner Sub-Workflow

**Figure 5:**
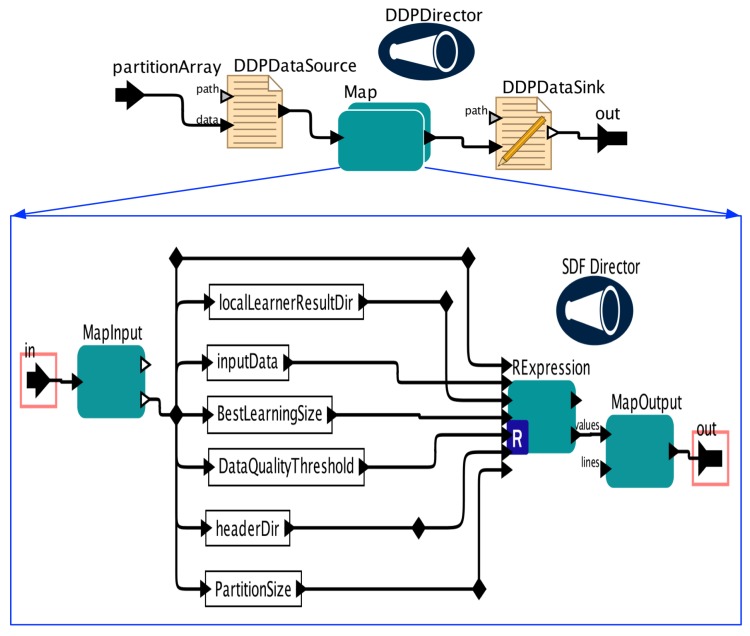
Local Learner Sub-workflow.

Local Learner is a composite actor whose sub-workflow is shown in Figure 5. Map DDP actor is used here to achieve parallel Local Learner execution. DDP Director is used to manage the sub-workflow execution by communicating with the underlying DDP engines. DDPDataSource actor reads global data slices generated by PartitionData actor and sends each global data slice to a Local Learner instance that runs across the computing nodes. The sub-workflow of the Map actor, shown in the lower part of the figure, mainly calls a RExpression actor to run the Local Learner R script shown in Algorithm 4.

### 6.4. Global Ensemble Sub-Workflow

**Figure 6:**
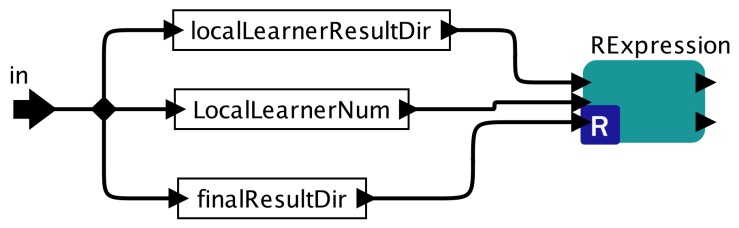
Global Ensemble Sub-workflow.

The sub-workflow of the Global Ensemble actor is shown in Figure 6. It mainly calls a RExpression actor to run the GlobalEnsemble R script (Algorithm 5) based on user-specified parameters. The Kepler engine manages their executions so that the GlobalEnsemble actor can only be executed after the Map actor finishes all Local Learner processing.

In summary, this workflow demonstrates how Kepler can facilitate building parallel network learner algorithms. The DDP framework of Kepler provides the basic building blocks for the DDP patterns and supports the dependencies between them. The RExpression actor can easily integrate user R scripts with other parts of the workflow. Kepler also provides subsidiary actors, such as Expression and DDPDataSource, for minor operations needed for a complete and executable workflow. Overall, a Kepler user can build scalable network learner workflows without writing programs, except for the core analytic scripts.

## 7. Experimental Setup

Hardware specification, datasets, and experimental setups are described in this section, and experimental results are presented in the next section.

### 7.1. Hardware Specification

The machine specification for the evaluation of all results is as follows. Two to sixteen compute nodes in a cluster environment are employed, where each node has two eight-core 2.6 GHz CPUs, and 64 GB memory. Each node can access the input data via a shared Lustre parallel file system.

### 7.2. Datasets

We used three sets of datasets to evaluate performance in our experiments. The first set of datasets are generated by ALARM (A Logical Alarm Reduction Mechanism) network, is used to build a Bayesian network designed to provide an alarm message system for patient monitoring [51]. The second set of datasets, Child, is used to build a Bayesian network for diagnosing lung diseases [52]. The third set of datasets, Insurance, is used to build a Bayesian network for evaluating car insurance risks in the transportation domain [53]. Each dataset was generated with 10 million samples (denoted 10 M), 20 million samples (20 M), 50 million samples (50 M), 100 million (100 M), 150 million (150 M), and 200 million (200 M) samples. Ten datasets were generated for each size. Each of these datasets was independently generated by the SamIam tool [54]. The properties of three Bayesian networks are listed in Table 1, these three networks have distinct and different structures. The experiment datasets and their sizes are listed in Table 2.

### 7.3. PEnBayes Experimental Setup

For software, we used Kepler version 2.5 with bioKepler suite version 1.1 as add-on modules. We used Spark version 1.5.2 with scripts for starting Spark automatically and trigger Kepler workflow execution. For configuration, we set the number of worker processes per node of Spark, i.e., SPARK_WORKER_INSTANCES, to be 8, and the localLearnerNum and degreeOfParallelism parameter of Kepler to be half of the total available core number. It is based on our findings that half the number of the available core can achieve the best performance when executing legacy tools on top of the big data platform [13]. The thresholds $\epsilon_{1}$ and $\epsilon_{2}$ used in Algorithm 1 are set to be 0.05 for calculating ALS. BDeu score [29] is used throughout the experiment as the Bayesian score. We choose 0.05 as the threshold based on the experiment conducted in Section 8.1. The data set is stored in the data server, and is accessed by the local learner through DataFrames of Spark and R.

### 7.4. Baseline Experimental Setup

To quantify the benefits of PEnBayes for Bayesian network learning on big data, we conducted a series of baseline experiments using conventional learning algorithms to construct Bayesian networks. The R package bnlearn [55] was used to implement these algorithms, and the three algorithms used were MMHC, HC, and Tabu.

The baseline experiments were executed on the same machine described in Section 7.1. The setup was different, however, in that each baseline experiment was run serially on a single node since there is no parallelism in conventional Bayesian learning algorithms. Each of the three algorithms (MMHC, HC, and Tabu) was used to learn a network for each of the datasets described in Section 7.2.

## 8. Experimental Results

This section presents experimental results with our PEnBayes approach. Section 8.1 discusses evaluation of the ALS calculation algorithms to determine the Appropriate Learning Size for big datasets. Section 8.2 compares execution time and accuracy for PEnBayes with the baseline algorithms for learning Bayesian networks. Section 8.3 explores the scalability of PEnBayes. Note that PB16, PB32, PB64 and PB128 refer to results of PEnBayes with 16, 32, 64 and 128 Local Learners, respectively. Based on the setup in Section 7.3, they each run with 32, 64, 128 and 256 CPU cores.

### 8.1. ALS Calculation Results

Table 3 shows the computation results for Appropriate Learning Size (ALS) and comparison between the calculated AMBS and actual AMBS from the three known BNs. We observe that the calculated AMBS by Algorithm 1 is very close to the actual AMBS, indicating an accurate estimation of ALS by Algorithm 1.

**Figure 7:**
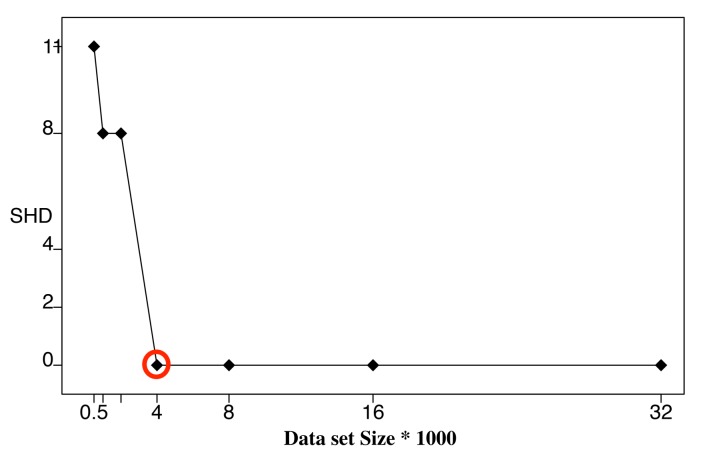
Structural Hamming distance (SHD) of different data set sizes using calculated ALS (Table 3) as reference value (red circle), Child Dataset.

**Figure 8:**
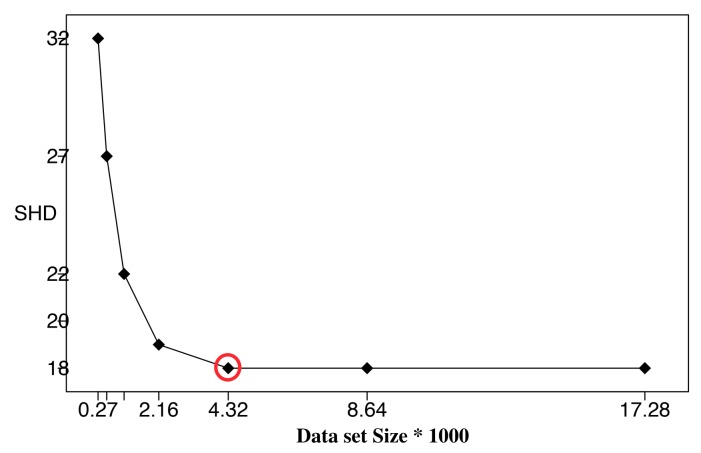
Structural Hamming distance (SHD) of different data set sizes using calculated ALS (Table 3) as reference value (red circle), Insurance Dataset.

**Figure 9:**
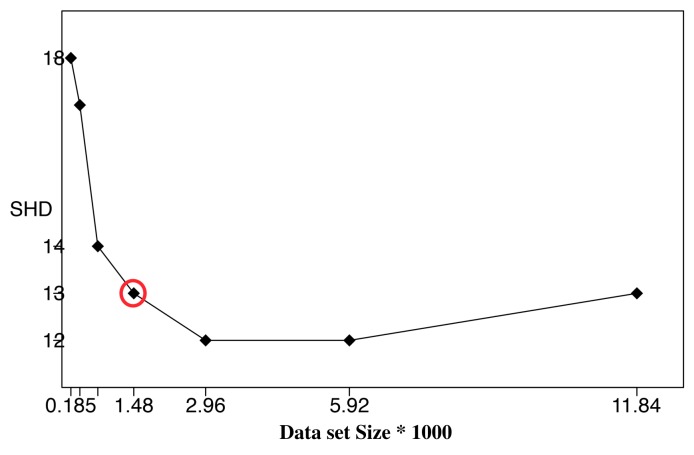
Structural Hamming distance (SHD) of different data set sizes using calculated ALS (Table 3) as reference value (red circle), Alarm Dataset.

To further verify the correctness of the ALS calculation, we performed BN learning using the calculated ALS values (the second column in Table 3) as reference values (circled in red). Each reference value was repeatedly halved or doubled to create a new dataset size. For each dataset size, a network was learned using the HC algorithm, and the resulting structural Hamming distance (SHD) between the learned network and the correct network was recorded [27]. We only use SHD because it can distinguish the differences between the learning results clearly. Bayesian score comparison results are much closer, and differences in the learned network are hard to identify and distinguish. Figure 7, Figure 8 and Figure 9 show SHD trends over varying data slice size on the three synthetic datasets. The reference values are circled in red.

Notice that a lower SHD value indicates a more accurate BN structure. From the figures, we observe that SHD drops sharply as the data slice size increases until the reference value of ALS is reached. We also notice that as the data slice size increases from the reference value, SHD remains stable. In Figure 9, the calculated ALS does not achieve the lowest SHD. Further, SHD increases as data size increases, indicating the existence of overfitting with the growth of the data set size. However, we observe that the SHD value of the calculated ALS is 13, which is very close to the lowest SHD value being 12. This indicates that the calculated ALS is the smallest data slice size that returns the lowest or close to lowest SHD values, thus optimizing the trade-off between learning accuracy and computation efficiency and avoiding overfitting. These results show the effectiveness of Algorithm 1 for calculating ALS. This empirical study also supports setting the value of thresholds $\epsilon_{1}$ and $\epsilon_{2}$ as 0.05.

### 8.2. PEnBayes and Baseline Experimental Result Comparison

Execution time, as well as accuracy, are compared for different PEnBayes setups (with different numbers of Local Learners) and different baseline Bayesian learning algorithms, namely MMHC, HC, and Tabu.

**Figure 10:**
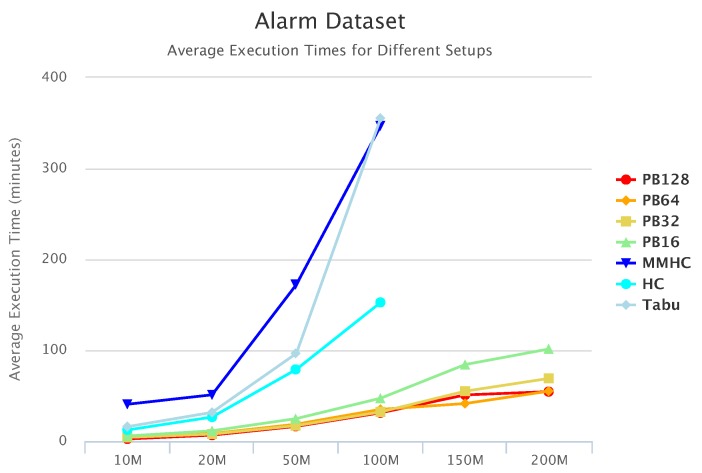
Alarm Set Execution Time.

**Figure 11:**
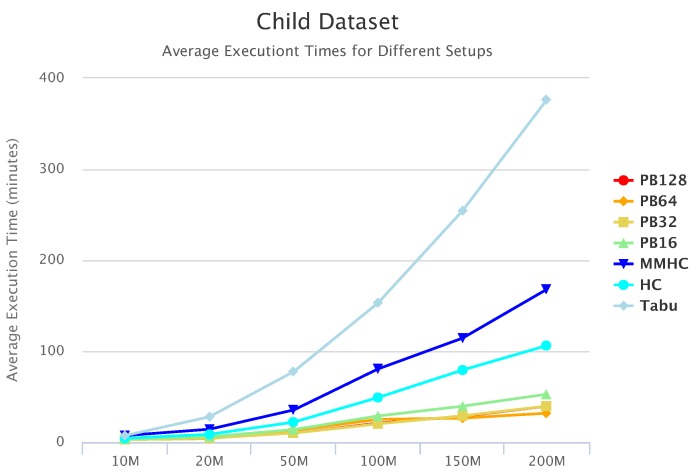
Child Set Execution Time.

**Figure 12:**
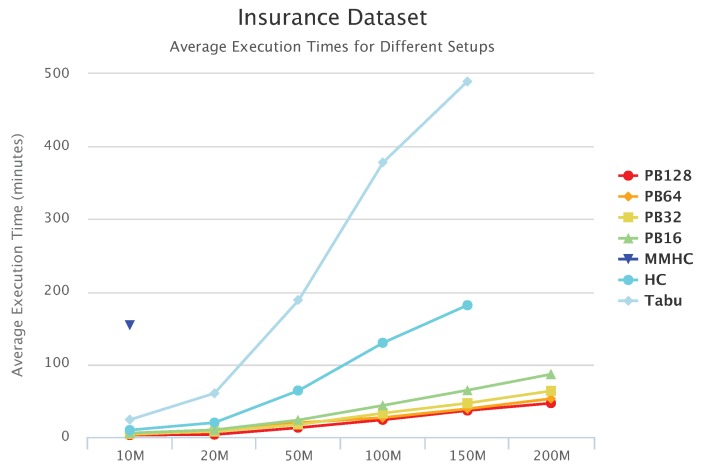
Insurance Set Execution Time.

Figure 10, Figure 11 and Figure 12 compare execution times of the different setups for the Alarm, Child, and Insurance datasets, respectively. As these figures show, execution times for the baseline algorithms are exponentially higher than for PEnBayes and increase at a much faster rate as the dataset size increases. Note that for the Insurance dataset, MMHC was able to complete learning for only the 10-M dataset. For all other Insurance dataset sizes, MMHC was not able to learn the network within the maximum allotted amount of 12 h for the wall-time. These results demonstrate the significant improvement of computational efficiency of PEnBayes compared with traditional baseline BN learning algorithms.

**Figure 13:**
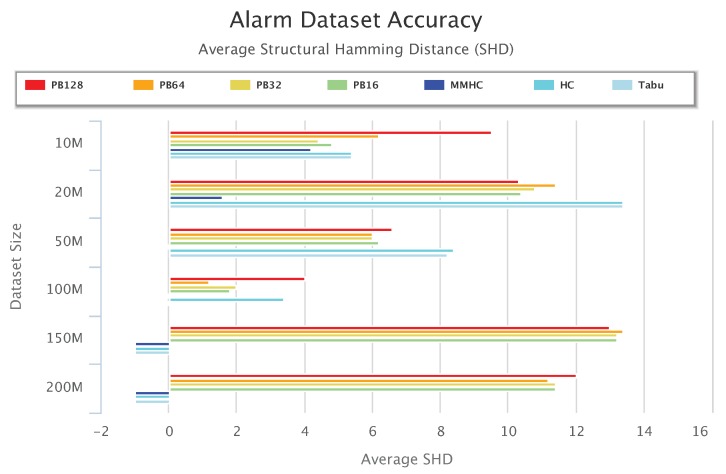
Alarm Dataset Accuracy Results. Negative values indicate that the algorithm was unsuccessful in learning a network for the dataset.

**Figure 14:**
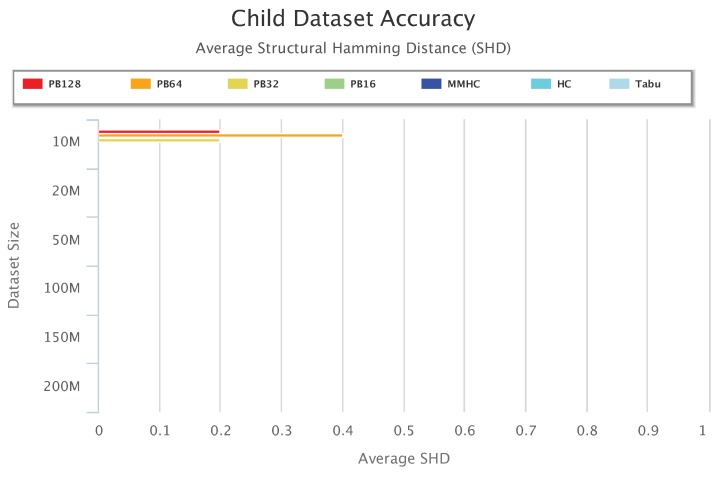
Child Dataset Accuracy Results.

**Figure 15:**
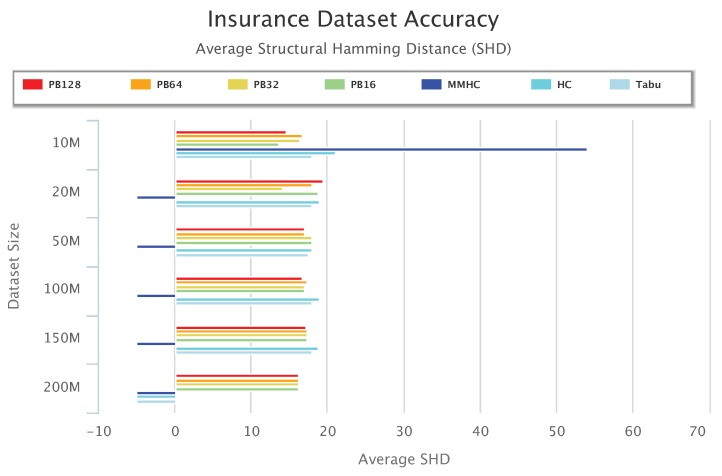
Insurance Dataset Accuracy Results.

Figure 13, Figure 14 and Figure 15 compare accuracy of the different setups for the Alarm, Child, and Insurance datasets, respectively. Accuracy is measured by the structural hamming distance (SHD) between the learned network and the true network structure. Lower SHD indicates a more accurate BN structure. In these figures, a value of zero for SHD indicates that the learned network exactly matches the true network. A negative value for SHD, on the other hand, indicates that network learning was not completed within the maximum allotted amount of 12 h for the wall-time.

Figure 13 shows that for the Alarm 50-M dataset, MMHC was able to identify the structure of the true network. For the Alarm 100 M dataset, both MMHC and Tabu were able to achieve this. However, for the Alarm 150-M and 200-M datasets, none of the baseline learning algorithms was able to learn a network at all within the maximum allotted time. Only PEnBayes was able to learn a BN structure from these very large datasets. Among the baseline algorithms, MMHC achieved the best learning accuracy. On 20-M, 50-M and 100-M datasets, PEnBayes achieves better accuracy than whole dataset learning by HC and Tabu algorithms. This indicates the effectiveness and robustness of PEnBayes in obtaining more consistent and accurate BN structures through multi-layered ensembles.

Figure 14 shows accuracy results for the Child data. All setups achieved a perfect SHD value of zero, except for PB128, PB64, and PB32 on 10 million datasets. Note that PB16 achieved 100% learning accuracy with 0 SHD.

Figure 15 summarizes accuracy results for the Insurance data. Note that for Insurance 200 M, none of the baseline algorithms was able to learn a network. An important observation from this plot is that MMHC performed very poorly on the Insurance data. MMHC learned a network with very high SHD for Insurance 10 M, and was not able to complete network learning for all other Insurance datasets. However, with the multi-layered ensemble approach, PEnBayes was not affected by the poor performance of MMHC. In contrast, Figure 15 shows that PEnBayes achieved a slightly higher learning accuracy than all baseline algorithms, with smaller SHD values on all Insurance datasets.

We also noted that for the same big dataset, different numbers of Local Learners may result in different learning accuracies. This is because the Bayesian networks learned by the Local Learners are different in number and structurally varied, thus resulting in different final network structures, and consequently, slightly different accuracy results. In addition, we observe that there is no optimal number of Local Learners for learning accuracy in general.

**Figure 16:**
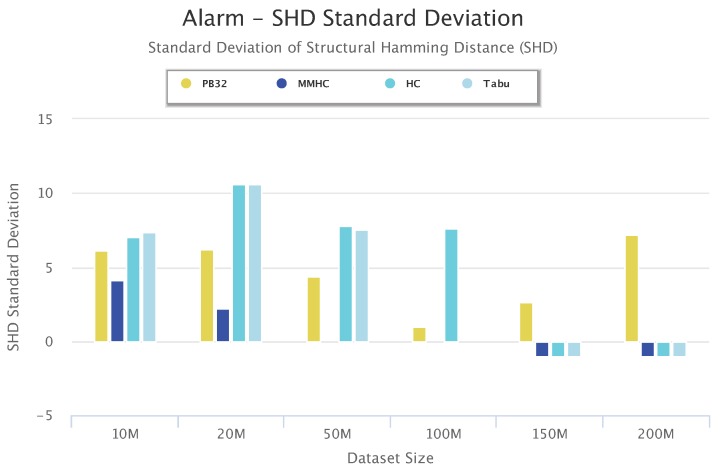
Alarm Set Standard Deviation Results.

To study the accuracy and the stability of PenBayes, we compared the SHD standard deviation of PenBayes vs. the baseline algorithms. Figure 16 shows the standard deviation of the SHD for Alarm for different dataset sizes. As with previous figures, negative values indicate that the algorithm was unsuccessful in learning a network for the dataset. We observe that even though the SHD standard deviation of PenBayes was not always the lowest, it was more consistent across all dataset sizes compared to the baseline algorithms. For example, even though MMHC obtained lower SHD standard deviation values than PenBayes for the smaller datasets, it was not able to learn a network for dataset sizes 150-M and 200-M. Tabu had higher SHD standard deviation values than PenBayes for dataset sizes 10 M to 50 M, then abruptly dropped to 0 for 100-M, and was not able to process dataset sizes 150-M and 200-M. This indicates that PenBayes achieves more stable learning accuracy than any individual baseline algorithm alone.

In summary, compared with the baseline learning algorithms, these experimental results indicate that PEnBayes is more stable and robust regarding learning BN structures from big datasets than traditional learning algorithms.

### 8.3. PEnBayes Scalability Experiments

**Figure 17:**
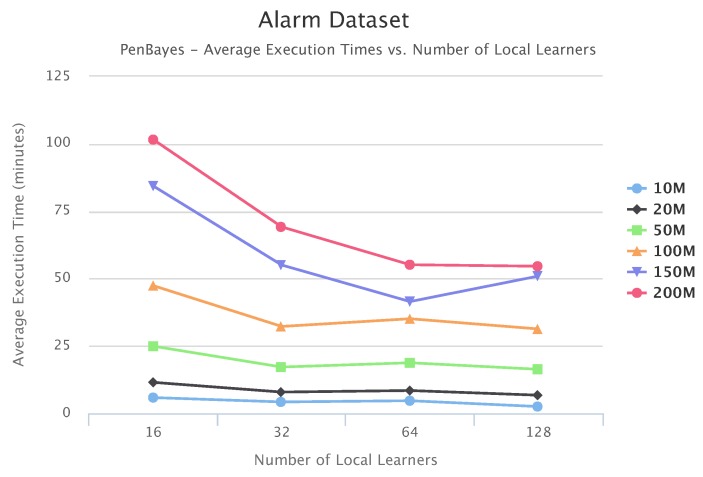
Alarm Set Execution Time vs. Number of Local Learners.

**Figure 18:**
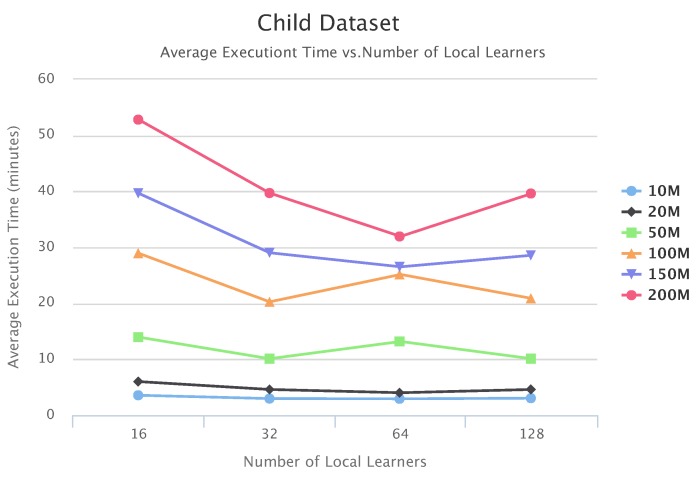
Child Set Execution Time vs. Number of Local Learners.

**Figure 19:**
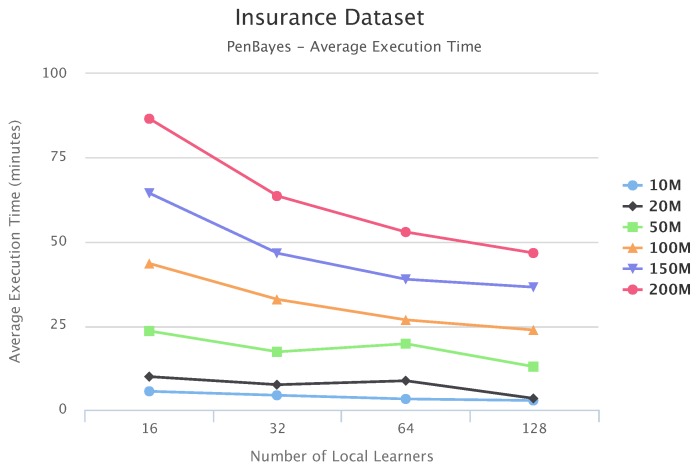
Insurance Set Execution Time vs. Number of Local Learners.

Figure 17, Figure 18 and Figure 19 show the scalability of our workflow with different Local Learner number and distributed nodes. We always allocate eight Local Learners on one compute node. Therefore, more Local Learner means the execution runs on more compute nodes. We can see that most of the execution times decreased with more Local Learners, showing a linear relationship between the number of Local Learners and the execution time. This indicates that PenBayes can scale well, achieving better execution performance with more computation nodes.

We observe that there were a few cases where the execution times increased with more Local Learners. We investigated the logs on these cases and found that while more Local Learners decreased learning time for each Local Learner, data reading times by the Local Learner program were nearly constant. In the experiments, data was read from a shared data node with a Lustre parallel file system. The reason for the constant data reading time is because more Local Learners will have fewer data to be read for each Local Learner, but more reading competition among more simultaneous data readers. This means that data reading became more and more dominant in the overall execution time. Also, the same amount of data sent to the Local Learners does not mean the same local learning time because learning time, especially its convergence, depends on both data content and data size. Data content affects how samples for different dependency relationships are distributed among different data slices. Therefore, if the jobs scheduled on one compute node by Spark happen to have more data reading time and learning time, the node becomes a straggler [56] and slows down the overall execution time. Additionally, these experiments were conducted in a shared cluster environment where network congestion varies depending on the load. Due to these reasons, we do not always see the overall times decrease with more Local Learners. This abnormal behavior occurred more with the Child datasets since their learning times are relatively shorter compared to Alarm and Insurance.

## 9. Conclusions

In this paper, we propose a novel parallel learning approach called PEnBayes (Parallel Ensemble-based Bayesian network learning) for a learning Bayesian network (BN) structure from big data. PEnBayes contains a novel greedy data size calculation algorithm for adaptively partitioning a big dataset into data slices of appropriate size for distributed BN learning. It achieves a stable and accurate Bayesian network learning from big datasets in a distributed multi-layer ensemble fashion at both data and algorithm levels.

Experimental results on big datasets generated by simulating sensor big data from patient monitoring, transportation, and disease diagnosis domains demonstrate that PEnBayes brings significant improvements in execution time compared with whole dataset learning. Moreover, PEnBayes achieves more consistent and robust Bayesian network structure learning than baseline algorithms. PEnBayes enables big data Bayesian graphical modeling and makes algorithm selection and learning results integration automatic when performing BN structure learning tasks. Our future work will focus on enriching the ensemble of learning algorithms [57] and making the local learning more adaptive and distributed to achieve higher earning accuracy and efficiency. Also, causality, interpretability as part of explainable AI [58] is of utmost importance for future research.

## Figures and Tables

**Table 1:** Bayesian Networks.

Name	Nodes	Edges	AMBS	Edge Strength
Alarm	37	46	3.51	0.23
Child	20	25	3.0	0.49
Insurance	27	52	5.19	0.25

**Table 2:** Data set sizes (unit: GB).

Dataset	10 M	20 M	50 M	100 M	150 M	200 M
Alarm	1.828	3.656	9.140	18.280	27.421	36.561
Child	1.073	2.146	5.364	10.728	16.093	21.457
Insurance	1.829	3.658	9.144	18.288	27.432	36.576

**Table 3:** ALS study results.

Network	Calculated ALS	Calculated AMBS	Actual AMBS
Alarm	14,800	3.656	3.51
Child	4000	3.00	3.00
Insurance	43,200	4.66	5.19

## Abbreviations

The following abbreviations are used in this manuscript:

BN	Bayesian Networks
PEnBayes	Parallel Ensemble based Bayesian network learning
MB	Markov Blanket
ALS	Appropriate Learning Size
DAG	directed acyclic graph
DDP	Distributed Data-Parallel
UDF	User Defined Functions
GUI	Graphical User Interface
BDeu	Bayesian Dirichlet equivalence with uniform prior
UDF	User Defined Functions
HC	Hill Climbing
TPDA	Three Phase Dependency Analysis
MMHC	Max-Min Hill-Climbing
AMBS	Average Markov Blanket Size
ES	Edge Strength
FWAM	Final Weighted Adjacent Matrix
AMBS	Average Markov Blanket Size
ALARM	A Logical Alarm Reduction Mechanism
SHD	Structural Hamming distance
